# Reelin Alleviates Mesenchymal Stem Cell Senescence and Reduces Pathological α-Synuclein Expression in an In Vitro Model of Parkinson’s Disease

**DOI:** 10.3390/genes12071066

**Published:** 2021-07-13

**Authors:** Eunju Cho, Joonsang Park, Kyungri Kim, Min-Gi Kim, Sung-Rae Cho

**Affiliations:** 1 Department and Research Institute of Rehabilitation Medicine, Yonsei University College of Medicine, Seoul 03722, Korea; bioneun00@yonsei.ac.kr (E.C.); jsmspark@naver.com (J.P.); kby930@yuhs.ac (K.K.); mg0521k@naver.com (M.-G.K.); 2 Brain Korea 21 PLUS Project for Medical Science, Yonsei University College of Medicine, Seoul 03722, Korea; 3 Graduate Program of Nano Science and Technology, Yonsei University College of Medicine, Seoul 03722, Korea; 4 Rehabilitation Institute of Neuromuscular Disease, Yonsei University College of Medicine, Seoul 03722, Korea

**Keywords:** parkinson’s disease, transcriptome analysis, extracellular matrix-receptor related genes, Reelin

## Abstract

Parkinson’s disease (PD) is one of the most common neurodegenerative diseases. The mechanisms underlying PD remain to be fully elucidated, and research into treatments for this condition is ongoing. Recent advances in genetic research have shed light on the mechanisms underlying PD. In this study, we used PD and control mesenchymal stem cells (MSCs) obtained from adipose tissues to confirm the differences between groups at the cellular and molecular levels. The results revealed that in PD MSCs, cell viability was clearly lower, and the rate of cell senescence was higher compared to the controls. Next, to compare the gene expression in PD and control cells, transcriptome analysis was performed. Genes in pathways, including extracellular matrix (ECM) receptor interaction, P53 signaling, and focal adhesion, were down-regulated in PD. Among genes related to ECM receptor interaction, *RELN* gene expression was markedly decreased in PD cells; however, after being treated with recombinant Reelin protein, a significant increase in cell viability and a decrease in α-Synuclein aggregation and cell senescence were observed. In conclusion, Reelin affects PD by positively influencing the cell characteristics. Our findings will facilitate research into new treatments for PD.

## 1. Introduction

Parkinson’s disease (PD) is a neurodegenerative disorder caused by the gradual deterioration of midbrain dopaminergic (DA) neurons in the substantia nigra pars compacta. Changes in DA cells lead to tremor and rigidity, and induce a slow decline of the extrapyramidal motor system. Despite this, treatments for PD and its underlying pathogenesis remain understudied [1]. The main pathogenic effect of PD is the degeneration of DA neurons in the substantia nigra pars compacta; however, there is evidence that tissues in other physiological systems contribute to the symptoms of PD [2]. Gene expression profiling (GEP) research has consistently detected problems with mitochondria or protein processing in PD, revealing potential new biomarkers for the disease. Previous studies conducted a comparison of the biological pathways identified from microarray study data, which was an effective method to solve interstudy variability in gene level analyses [3,4]. Postmortem GEP studies conducted on the substantia nigra of PD patients have implicated the number of biological pathways, which also function in non-nigral tissues [5].

Neurodegeneration in non-nigral tissues contributes to the non-motor symptoms of PD, which include dementia and depression. Dopamine replacement therapy is less effective in combating these non-motor symptoms relative to motor symptoms [6,7]. Therefore, research on the neuronal degeneration of non-nigral tissue has potential, not only to deepen the understanding of PD pathogenesis, but also to provide information useful for the development of treatment for non-motor symptoms.

Mesenchymal stem cells (MSCs) are multipotent cells that can differentiate into neuron-like cells, and they have been studied as part of potential therapy for PD [8,9]. MSCs are also used to determine differential gene expression and clustering analysis via transcriptome analysis without any ethical issues and low donor-site morbidity so that adipose-derived MSCs can be used as promising candidates for various clinical applications, representing human cell model [8,10,11]. However, MSCs get limited regenerative capacity depending on the age, and it has low survival rate when used in PD patients as replacements for disease neurons in the host [12,13,14]. We questioned what the differences would be in molecular and cellular levels between the control and PD patient MSCs, and how to increase the survival rate of MSCs. Since PD is an age-related neurodegenerative disease associated with systemic problems, including inflammation, MSCs isolated from the abdominal adipose tissue were used in our cell model for PD to conduct GEP investigation in this study [15]. Our results provide important data pertinent to research of the mechanisms underlying PD and potential treatment targets [16]. Particularly, our study investigated the biological pathway associated with extracellular matrix (ECM) found by transcriptome analysis in PD. There are studies showing that ECM molecules and their receptor expression level alternations are associated with central nervous system diseases, including the human brain aging with PD [17,18,19]. Transcriptional- and translational-level regulation of ECM-degrading enzymes, such as matrix metalloproteinases, can affect disease pathogenesis [18]. Similarly, our research using transcriptome analysis to examine specific changes at the gene expression level identified ECM-associated pathways as implicated in PD.

## 2. Materials and Methods 

### 2.1. Subjects 

This study was approved by the Ethics Committee, and the participants signed informed consent prior to the study. Human adipose-derived MSCs were obtained from a 68-year-old female volunteer as a control; moreover, the patient-derived samples were obtained with the agreement of the participants, who provided his or her written informed consent. The Institutional Review Board of Severance Hospital, Yonsei University Health System approved this consent procedure and the entire study (no. 4-2008-0277, 4-2012-0028). An age-matched female patient with PD, aged 68 years, who was not receiving any pharmacological treatment, was included in this study. The patient was recruited at the Rehabilitation Institute of Neuromuscular Disease. The patient with PD included in this study did not show any signs of infection before undergoing liposuction. 

### 2.2. Cell Culture

MSCs were cultured from the abdominal adipose tissue samples from a control patient and patient with PD. The biopsy sample was transferred to a culture dish in low glucose Dulbecco’s modified Eagle’s medium (DMEM) containing 10% fetal bovine serum (FBS) and penicillin/streptomycin (P/S). For human neuroblastoma cells, SH-SY5Y cells were cultured with Dulbecco’s Modified Eagle Medium/Nutrient Mixture F-12 (DMEM/F12) containing 10% FBS and 1% P/S. Cells were incubated in a humidified 5% carbon dioxide atmosphere at 37 °C. 

### 2.3. Cell Viability and Senescence Analysis 

For analysis of cell viability, MSCs were seeded in 6-well plates (10^3^ cells/well) in DMEM containing 10% FBS and P/S. The number of cells per plate was determined from counts obtained using an ADAM automatic cell counter 2, 4, 6, and 8 days after plating, as described in the manufacturer’s protocol (NanoEnTek Inc., Seoul, Korea). Flow cytometric analysis of cellular senescence was performed using a Quantitative Cellular Senescence Assay Kit (Cell Biolabs, San Diego, CA, USA). Briefly, MSCs were treated with pretreatment solution at 37 °C for 2 h. Next, senescence-associated β-galactosidase (SA-β-gal) substrate solution was added to the cells for 4 h. Stained cells were washed with Dulbecco’s phosphate-buffered saline (DPBS) and harvested by trypsinization. For microscopy studies, MSCs were washed with DPBS, fixed for 15 min in fixing solution at room temperature, briefly washed in DPBS, and then incubated with SA-β-Gal substrate solution at 37 °C without carbon dioxide and with protection from light for 16 h. Blue-stained cells were analyzed by light microscopy.

### 2.4. RNA Preparation

Total RNA was isolated from the cultured MSCs obtained from a patient with PD and controls using Trizol (Invitrogen Life Technologies, Carlsbad, CA, USA) according to the manufacturer’s instructions [20]. RNA purity was evaluated using an Agilent 2100 Bioanalyzer (Agilent Technologies, Palo Alto, CA, USA), and the A260/A280 ratio was measured for quality control analysis. RNA integrity was evaluated by visual evaluation of the 28S:18S rRNA ratio using gel electrophoresis.

### 2.5. RNA Sequencing Transcriptome Analysis 

RNA sequencing (RNA-seq) and transcriptome data analysis were performed by Macrogen Inc. (Seoul, Korea). mRNA libraries for cluster generation were prepared using the reagents provided in the Illumina TruSeq™ RNA Sample Preparation Kit [21]. Transcriptome analysis comprised RNA-seq experiments and data handling [22]. RNA-seq experiments involved TruSeq mRNA library construction, which included eight steps: (1) purify and fragment mRNA, (2) synthesize first strand cDNA, (3) synthesize second strand cDNA, (4) perform end repair, (5) adenylate 3’ ends (i.e., add a single adenosine residue), (6) ligate adapters, (7) enrich DNA fragments, and (8) validate the enriched library. First, poly-Adenine containing mRNA molecules were purified using poly-Thymine oligonucleotide-conjugated magnetic beads. After purification, using divalent cations at raised temperature (94 °C, 8 min), the mRNA was sheared into small fragments. First strand cDNA was generated from the cleaved RNA fragments using random hexamer primers and reverse transcriptase. The cDNA fragments were then subjected to an end repair process using an end repair mix, which added a single “Adenine” nucleotide, followed by adapter ligation. On the 3′ end of the adapter, there was a corresponding single “Thymine” nucleotide, which provided a paired overhang for binding to the adapter to the fragment. To create the final cDNA library, the products were purified and enriched by polymerase chain reaction (PCR) [23].

Illumina uses a unique reaction, referred to as “bridged” amplification, which occurs on the surface of the flow cell. The flow cell, which contains millions of unique clusters, is loaded into the HiSeq 2000 for automated cycles of imaging and extension. Solexa’s Sequencing-by-Synthesis utilizes four proprietary nucleotides with reversible fluorophore and termination properties. Every individual sequencing cycle occurs in the presence of all four nucleotides, leading to superior accuracy relative to methods in which only one nucleotide is present in the reaction mix [24].

To evaluate expression levels, RNA-seq reads were mapped to the human genome and transcript counts were calculated at the gene level. Data analysis was performed using TopHat and Cufflinks. TopHat is a fast splice junction mapper for RNA-seq reads. Using an ultra-high-throughput short read aligner known as Bowtie, TopHat aligns the RNA-seq reads to mammalian-sized genomes. Subsequently, TopHat analyzes the mapping results to identify the splice junctions between exons. Cufflink tests for differential expression and regulation in RNA-seq samples, in addition to assembling transcripts and estimating their abundance. Transcripts with fold induction <2 and Benjamini-Hochberg adjusted *p*-values < 0.05 were considered significantly different between control and PD samples, and were included in the downstream analysis.

### 2.6. Kyoto Encyclopedia of Genes and Genomes Pathway Analysis 

For analysis, the database for annotation, visualization, and integrated discovery (DAVID) software (http://david.abcc.ncifcrf.gov/, accessed on 25 November 2015) [25] and Kyoto Encyclopedia of Genes and Genomes (KEGG) pathways [26,27] were selected. Using the DAVID software, our study found which pathways were down-regulated in PD cell.

### 2.7. Reverse Transcription and Quantitative Reverse Transcription Polymerase Chain Reaction 

This study additionally performed reverse transcription polymerase chain reaction (RT-PCR) to validate the results from the transcriptome analysis. The RT-PCR of genes were components of significantly down-regulated KEGG pathways, which is related to neuronal protection. Quantitative real-time polymerase chain reaction (qRT-PCR) was conducted, as described in detail in a previous study [28]. For additional validation, qRT-PCR was performed in triplicate on a Light Cycler 480 (Roche Applied Science, Mannheim, Germany) using the Light Cycler 480 SYBR Green master mix (Roche Applied Science), and thermocycler conditions of 10 min template preincubation step at 95 °C, followed by 40 cycles at 95 °C for 10 s, 60 °C for 10 s, and 72 °C for 10 s, followed by melting curve analysis, beginning at 95 °C for 5 s, followed by 1 min at 60 °C. The following primers were used for qRT-PCR: human *RELN* forward, 5′- TAC AGC GTC AAC AAC GGG AT-3′; *RELN* reverse, 5′- CGA CCT CCA CAT GGT CCA AA -3′ (190 base pair); human *GAPDH* forward, 5′- AAG GGT CAT CAT CTC TGC CC -3′; human *GAPDH* reverse, 5′-GTG AGT GCA TGG ACT GTG GT-3′ (180 base pair). The specificity of amplification products was confirmed by melting curve analysis, where all samples generated a distinct single sharp peak with the expected melting temperature. A distinct single peak indicates that a single DNA sequence was amplified during qRT-PCR. The glyceraldehyde 3-phosphate dehydrogenase (GAPDH) gene was used as the internal control. The expression level of each gene of interest was determined using the 2−ΔΔCt method. Specific primers were used for both RT-PCR and RT-qPCR (Table 1).

### 2.8. Western Blot Analysis

For immunoblot analysis, the cells were collected and lysed with lysis buffer, consisting of 10 mM Tris-HCl pH 7.4, 150 mM NaCl, 5 mM EDTA, 0.5% NP-40, Protease inhibitor cocktail (Cell signaling technology, Beverly, CA, USA), and Phosphatase inhibitor cocktail (GenDEPOT, Katy, TX, USA). The cell lysates were centrifuged at 18,000× *g* for 15 min. The supernatant was collected, and protein concentrations were determined by Bradford assay (Biorad, Hercules, CA, USA). Proteins were incubated in the anti-Reelin (Santacruz, Santa Rosa, TX, USA) for 4 h at 4 °C, following by the addition of protein G sepharose 4 fast flow beads (Sigma, Marietta, GA, USA) overnight at 4 °C. After that, the immunocomplexes were washed with lysis buffer, and then denatured by adding 2× LDS sample buffer (Invitrogen, Carlsbad, CA, USA), and boiled at 100 °C for less than 3 min. For immunoblotting, the samples were separated on SDS-PAGE gels and transferred to the PVDF (Millipore) membrane. Membranes were blocked for 1 h at room temperature in 5% bovine serum albumin or 5% skim milk in Tris-buffered saline with 0.1% Tween 20 detergent (TBST, Biosesang), and then incubated with primary antibodies against Reelin (Abcam) and β-actin (Santacruz) overnight at 4 °C. Membranes were washed in TBST and incubated with anti-mouse HRP-conjugated secondary antibody (Santacruz) for 1 h at room temperature. Membranes were washed in TBST and developed by the West-Q Pico ECL Solution (GenDEPOT). Chemi-luminescence signal was detected by the Chemi-iluminescence Image Analyzer (LAS), and determined using the Multi Gauge V3.0 software. Protein levels were normalized to β-actin levels from the same samples.

### 2.9. Preformed Fibril and Human Recombinant Reelin Treatment 

SH-SY5Y cells were differentiated with retinoic acid (RA, 10 μM, Sigma, Marietta, GA, USA) in high-glucose DMEM, containing 10% FBS and 1% P/S for 4 days. For preformed fibril (PFF) and human recombinant Reelin (hr-Reelin) treatment, differentiated SH-SY5Y cells were incubated with the medium, containing PFF (5 μg/mL, Stress Marq Biosciences Inc., British Columbia, CA, USA) and hr-Reelin (R&D systems, Minneapolis, MN, USA), for 4 days. For Thioflavin-S (Thio-S, Sigma) staining, SH-SY5Y cells were seeded onto poly-D-lysine (0.1 mg/mL, Sigma)-coated 15 mm coverglasses, and then cultured. On days in vitro 9 (DIV 9), fixed cells with 4% paraformaldehyde were incubated with 0.05% Thio-S for 8 min at room temperature. After three times of 5 min washing with 80% ethanol, the cells were rinsed three times with DPBS, and coverslipped using the antifade mounting medium with DAPI (VECTASHIELD).

### 2.10. Statistical Analysis 

Data are expressed as the mean ± standard deviation. Statistical analyses were performed using the Statistical Package for Social Sciences (SPSS) version 23.0 or GraphPad Prism (version 8.4.2, GraphPad software, La Jolla, CA, USA). Student’s *t*-test was used to evaluate the statistical significance of differences in human MSC data. One-way ANOVA followed by Bonferroni post hoc test was also used to determine the statistical significance of differences for SH-SY5Y cell data; *p*-values < 0.05 were considered statistically significant.

## 3. Results

### 3.1. Cell Viability Was Decreased in Parkinson’s Disease

**Figure 1:**
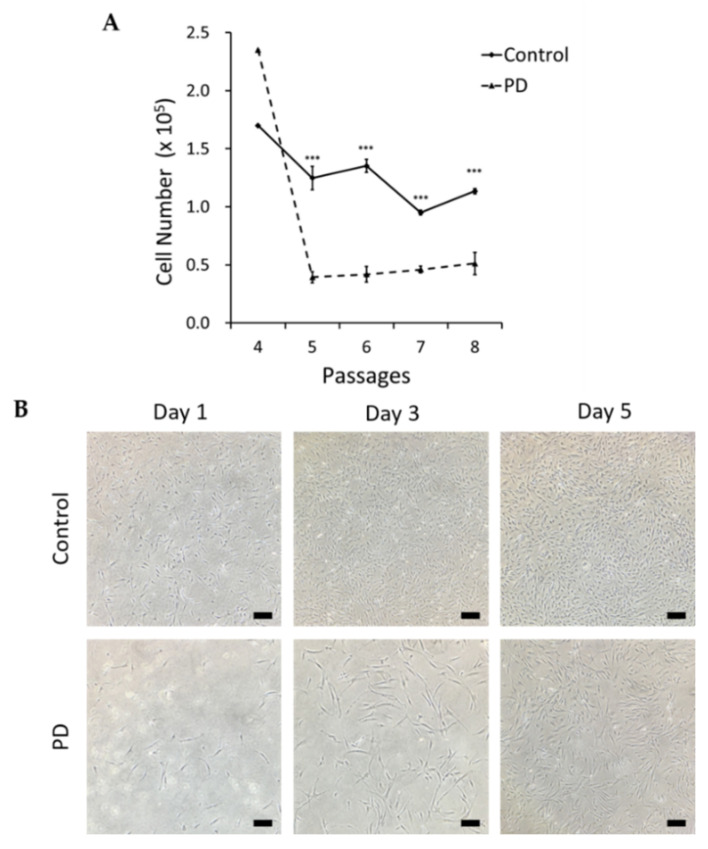
Comparison of cell viability in healthy control and Parkinson’s disease (PD) at each passage. (**A**) Growth curves for mesenchymal stem cells (MSCs) from a PD patient and healthy control at passages 4 to 8. *n* = 4 for each group. Mean ± S.E.M. (**B**) MSCs from control and PD subjects at passage 8 (Student’s *t*-test, *** *p* < 0.001), scale bars = 50 μm.

MSCs derived from a healthy individual and a patient with PD were cultured through passages 4 to 8 in 6-well plates, and the number of cells counted after each passage, to compare the proliferation rates between control and PD cells. Figure 1A shows that the number of PD cells decreased rapidly at passage 5, while the number of MSCs reduced more gradually in healthy control cells (Figure 1A). The numbers of PD cells were significantly decreased compared to the control cells from passages 5 through 8 (*p* < 0.001). Furthermore, differences between the proliferation of control and PD cells were clearly visible in serial cell images (Figure 1B and Figure S1).

### 3.2. Cell Senescence Was Increased in Parkinson’s Disease

**Figure 2:**
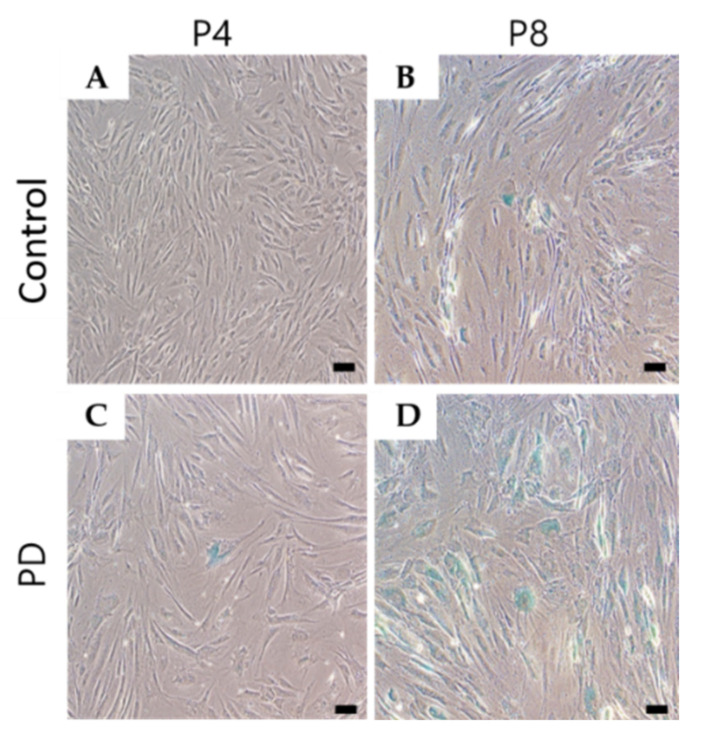
Comparison of cell senescence-associated β-galactosidase (SA-β-Gal) staining between control and Parkinson’s disease (PD) at passages 4 and 8 (**A**–**D**). SA-β-Gal activity in mesenchymal stem cells (MSCs) from a PD patient and healthy control. (**A**) MSCs from a control subject at passage 4. (**B**) MSCs from a control subject at passage 8. (**C**) MSCs from a patient with PD at passage 4. (**D**) MSCs from a patient with PD at passage 8. scale bars = 50 μm.

To compare the rates of cell senescence in each group, the number of cells stained blue, representing SA-β-Gal activation, was counted. Microscopy images showed that SA-β-Gal staining decreased in the control MSCs at passages 4 and 8 relative to PD cells (Figure 2A–D). In particular, cell senescence was increased at passage 8 in PD cells (Figure 2D).

### 3.3. Extracellular Matrix-Receptor Interaction-Related Genes Were Down-Regulated in Parkinson’s Disease

RNA samples were prepared from the cultured control and PD MSCs. To determine the influencing factors on the changes in cell viability and senescence, KEGG pathway analysis of candidate genes from transcriptome data was conducted. KEGG pathway results, generated using the DAVID program, identified which genes were involved in the pathogenesis of PD. Among the various pathways, including ECM receptor interaction, the P53 signaling, arrhythmogenic right ventricular cardiomyopathy, endocytosis, and focal adhesion were significantly enriched for genes dysregulated in PD (Table 1; *p* < 0.05). To elucidate the function of genes in the ECM-receptor interaction pathways, 10 down-regulated genes were selected for comparison of RNA expression patterns in the control and PD samples.

### 3.4. Both RELN mRNA and Reelin Protein Levels Were Down-Regulated in Parkinson’s Disease

**Figure 3:**
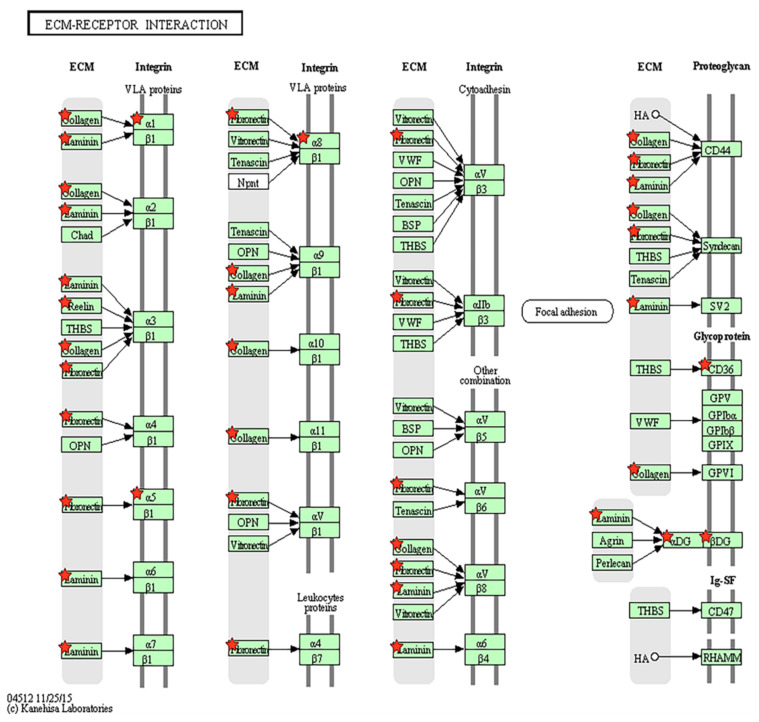
Illustration of extracellular matrix (ECM)-receptor interactions described in the Kyoto Encyclopedia of Genes and Genomes (KEGG) pathway. The KEGG pathway results generated using the DAVID program are shown above. Parkinson’s disease is associated with changes to ECM-related factors, including 10 selected down-regulated genes marked with red stars: *CD36*, *ITGA5*, *LAMA5*, *ITGA8*, *COL6A3*, *COL6A2*, *ITGA1*, *DAG1*, *RELN*, *COL5A3*.

Genes in the ECM-receptor interaction pathway were confirmed by analysis using the DAVID program. PD was associated with changes to ECM-related factors, including 10 selected down-regulated genes, were marked with red stars (Figure 3).

**Figure 4:**
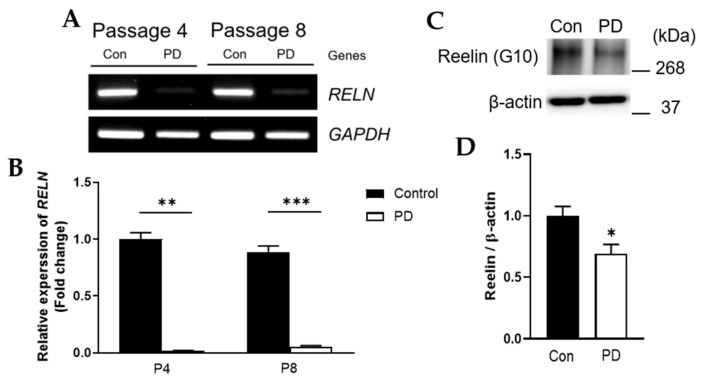
Validation of extracellular matrix (ECM)-related adhesion molecule *RELN* mRNA and Reelin protein levels. (**A**,**C**) Conformation of amplification using specific primer for *RELN* by RT-PCR (**A**) and Reelin protein by Western blot (**C**). (**B**,**D**) Quantifications of relative *RELN* expression by qRT-PCR (**B**) and Reelin protein level by Western blot (**D**). *n* = 3 (**A**,**B**) and *n* = 4 (**C**,**D**) for each group. Mean ± S.E.M. Student’s *t*-test, * *p* < 0.05, ** *p* < 0.01, *** *p* < 0.001. Parkinson’s disease, PD; Con, control.

RT-PCR of *RELN* mRNA and immunoblot for Reelin protein were subsequently performed for further validation. RNA and protein expression levels of Reelin appeared to be relatively low in PD compared to the control (Figure 4A,C). The selected gene and protein were evaluated by qRT-PCR and Western blot for more accurate quantitative analysis, respectively. RNA expression levels of *RELN* were significantly decreased at both passage 4 and 8 in PD compared to the control (Figure 4B, *p* = 0.002 and <0.001, respectively). The protein level of Reelin was also significantly decreased at passage 8 in PD compared to the control (Figure 4D, *p* = 0.028).

### 3.5. Recombinant Reelin Protein Regulates Cell Viability and Senescence 

**Figure 5:**
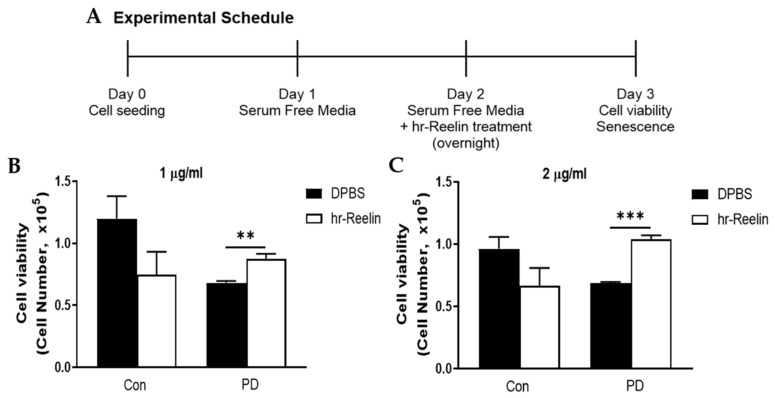
Cell viability at passage 8 following treatment with human recombinant Reelin (hr-Reelin) protein. (**A**) Experimental schedule and method for in vitro treatment with hr-Reelin proteins. (**B**,**C**) Viability of control and Parkinson’s disease (PD) cells. (**B**) Treatment with phosphate-buffered saline (DPBS) or hr-Reelin proteins at 1 μg/mL. (**C**) Treatment with DPBS or hr-Reelin proteins at 2 μg/mL. *n* = 5 for control group, *n* = 4 for PD group. Mean ± S.E.M. Student’s *t*-test, ** *p* < 0.01; *** *p* < 0.001.

To evaluate the effect of Reelin protein in both control and PD cells on cell proliferation, the experimental schedule in Figure 5A was followed, using two different protein concentrations. The hr-Reelin protein (1 μg/mL) also significantly induced proliferation of PD cells relative to DPBS treatment (Figure 5B, *p* = 0.0076). On culture with 2 μg/mL, hr-Reelin protein led to significant induction of cell viability compared with DPBS treatment (Figure 5C, *p* < 0.001).

**Figure 6:**
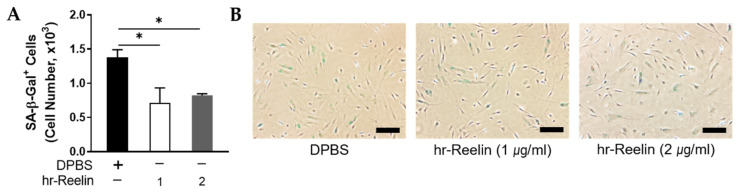
Cell senescence evaluated by β-galactosidase (SA-β-Gal) staining at passage 8 treated with human recombinant Reelin (hr-Reelin) protein in Parkinson’s disease (PD) patient MSCs. (**A**) SA-β-Gal-positive cells were compared between DPBS-treated group and 1 μg/mL or 2 μg/mL hr-Reelin-treated group. (**B**) Representative images. Scale bars= 50 μm. *n* = 6 for DPBS-treated group, *n* = 3 for hr-Reelin-treated groups. Mean ± S.E.M. Student’s *t*-test, * *p* < 0.05.

To test whether Reelin could inhibit PD MSCs from getting in replicative senescence-like state, known as the cell senescence, SA-β-Gal staining was performed and the cells stained in blue were counted (Figure 6). In the PD group treated with 1 μg/mL, hr-Reelin protein led to reduced SA-β-Gal-positive cells compared to the DPBS-treated control group (Figure 6A). At 2 μg/mL, PD cells treated with hr-Reelin protein showed significantly lower levels of senescence compared with DPBS-treated group control group. The hr-Reelin protein-treated group contained less SA-β-Gal-positive cells relative to DPBS-treated cells (*p* = 0.013); representative microscope images are presented in Figure 6B.

### 3.6. Recombinant Reelin Protein Attenuates α-Synuclein Aggragation in SH-SY5Y 

**Figure 7:**
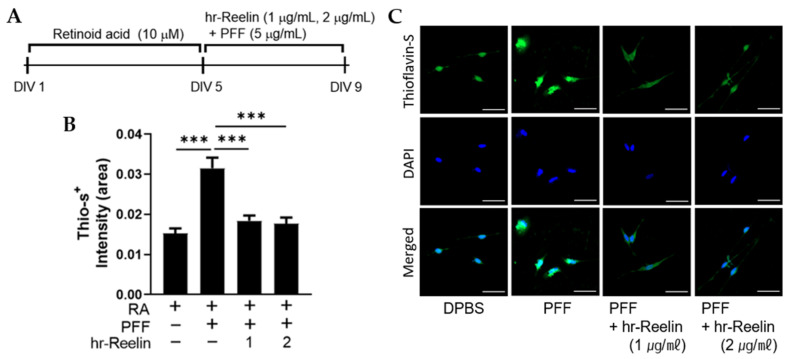
Aggregated α-Synuclein protein formation was measured by Thioflavin-S (Thio-S) staining in preformed fibril (PFF)-treated SH-SY5Y cells. (**A**) Differentiated SH-SY5Y cells were incubated with either PFF or human recombinant Reelin (hr-Reelin) protein (1 μg/mL and 2 μg/mL). (**B**) Each of Thio-S-positive intensities were compared between groups. (**C**) Representative images of (**B**). scale bars, 50 μm. Mean ± S.E.M. *n* = 52–95 cells in each group. One-way ANOVA (Bonferroni post hoc test), *** *p* < 0.001. Retinoic acid; RA, days in vitro; DIV.

To evaluate the effect of hr-Reelin protein, active α-Synuclein fibrils (PFFs) were added to the differentiated human neuroblastoma (SH-SY5Y) cells to induce the cellular model of PD (Figure 7A) [29,30]. Thioflavin-S (Thio-S) staining results showed that aggregated α-Synuclein (α-Syn) formation with PFF treatment were significantly attenuated by hr-Reelin protein treatment (either 1 μg/mL or 2 μg/mL) compared to the no hr-Reelin treatment group (Figure 7B,C).

## 4. Discussion

Senescence can be observed at the systemic and cell levels. Overall, senescence is associated with aging, and it also signifies a reduction in normal functions and an increased vulnerability to stress [31]. The concept of cell senescence was first introduced 50 years ago, based on the finding that cells in culture can undergo only a limited number of divisions [32]. Senescence is a process of change that generally occurs without apoptosis [33]. Cell senescence not only limits replicative ability but also changes cellular metabolism, epigenetic regulation, and gene expression. Molecular changes, such as alterations in morphology, growth factors and proteases, and cytokine secretion, that occur during cell senescence, are referred to as the senescence-associated secretory phenotype [34]. Cell senescence is related both to aging and to neurodegenerative diseases, such as Alzheimer’s disease (AD), PD, and frontotemporal dementia. Cell cycle arrest and cell senescence are known to be some of the main causes for PD, which can activate cyclin-dependent kinase (cdk5), induce neuronal inflammasomes, and lead neuronal cells to enter a senescence-like state or death in PD [35,36,37,38]. In addition, cell senescence affects ECM composition and content [39]. ASCs are a primary source of MSCs that are characterized by self-renewable and replicating cells. However, several studies have reported that after a certain number of serial subcultures, MSCs enter replicative senescence state and cease to proliferate [12,32,40,41].

This study identified the differences between PD and control cells. These findings support the theory that neurodegenerative disease is related to cell senescence and cell viability. By using the DAVID software, this study found that ECM-receptor related genes were characteristically down-regulated in PD cells. Through SA-β-Gal positive cell counting and Thio-S staining, our results suggest that the ECM molecule, Reelin, plays structurally and functionally important roles in maintaining the cell viability, attenuating cell senescence, and decreasing pathological α–Synuclein (α-Syn) levels. Based on our findings, in PD cells, problems with this function might have some influence on the decreased cell viability and difference in phenotype. Using RT-PCR and human recombinant protein experiments, this study found that *RELN*, among the ECM receptor-related genes, was particularly down-regulated by GEP investigation. 

Reelin is an ECM glycoprotein that controls brain development by regulation of neuronal migration and positioning [42]. Reelin also has an important role in brain development via its effects on neuronal migration, dendritogenesis, and synaptic transmission. Homozygous Reeler mice, lacking *RELN*, are severely ataxic and have structural anomalies in neocortical, hippocampal, and cerebellar structures [43,44]. The effects are less severe in heterozygous Reeler mice; however, these animals do have problems with dendrite development and cognitive function [45,46]. Reelin controls synaptic plasticity by influencing long-term potentiation both during development and in adulthood [47,48], and contributes to AD pathogenesis. Apolipoprotein E receptor 2 and very-low-density-lipoprotein (VLDL) receptor, which are Reelin receptors, belong to the low-density-lipoprotein (LDL) receptor gene family, and are referred to as the apolipoprotein E receptor [49]. Apolipoprotein E4, an apolipoprotein E isoform, has an important role in AD pathogenesis [49,50]. According to recent research, there are fewer Reelin-producing Cajal-Retzius cells in the first cortical layer in patients with AD [51,52], and Reelin contributes to AD pathogenesis by controlling synaptic glutamatergic transmission and potentiation [50,53]. Endocytosis and shuttling mechanisms of intracellular aggregations with α-Syn burden are important subject to study in PD. Many studies have focused on revealing the underlying mechanisms of impaired endocytosis and lysosomal rupture in PD [54,55,56]. We also found the relationship between α-Syn and vesicle associated membrane protein (VAMP2) [57]. However, the association of ECM molecule, particularly glycoprotein Reelin, with α-Syn burden still need to be explored further. Therefore, we selected ECM proteins to confirm whether they could be a promising therapeutic target in PD.

In the present study, using transcriptome analysis, the down-regulation of ECM receptor interaction-related genes was observed. Both RNA and protein expressions of Reelin were significantly decreased in PD compared to the control cells at passage 8. The differences in expression patterns determined by transcriptome analysis were confirmed by RT-PCR, qRT-PCR, and Western blot. MSCs derived from a control individual and a patient with PD were cultured from passage 4 to 8, during which the number of PD MSCs decreased rapidly. In contrast, the number of control MSCs decreased rapidly at passage 5. The SA-β-Gal staining of control MSCs at passages 4 and 8 was reduced compared to PD, indicating higher rates of senescence of PC cells. To evaluate the effect of Reelin in both control and PD cells, passage 8 MSCs from both groups were treated with various concentrations of hr-Reelin proteins. The results showed that Reelin could significantly induce proliferation and inhibit acceleration of cell senescence compared with DPBS controls.

Abnormal accumulation and aggregation of α-Syn proteins are neuropathological features of PD patients [58]. PFFs, which are aggregated form of α-Syn proteins, can induce cell senescence and cell death [30,59,60]. In this study, we tested whether Reelin protein could protect dopaminergic neurons from PFFs in SH-SY5Y cells model of PD. Thio-S staining analysis showed that hr-Reelin effectively suppressed the α-Syn aggregations that were induced by PFFs in SH-SY5Y cells. In line with these findings, we support the hypothesis that Reelin has a beneficial effect to neuron cells in PD patients. Our study found that Reelin could cause positive effects on ECM function, such as increasing cell viability, decreasing cell senescence, and suppressing the α-Syn aggregation. Importantly, these findings could assist in understanding PD pathogenesis and selecting treatment targets; but first, further study is needed to confirm whether Reelin ECM molecule also has beneficial effects in primary neuronal cell model and animal models of PD. Second, uncovering how Reelin ECM molecule is involved in enhancing cell viability, attenuating cell senescence, and reducing pathological α-Syn formation is also required in both primary neuronal cell model and animal model of PD.

## 5. Conclusions

In this study, the GEP of MSCs on both PD cells and control group was analyzed. *RELN* was among ECM-receptor-related genes whose expression was down-regulated in PD, and hr-Reelin prevented PFF-induced α-Syn aggregation. A better understanding of Reelin functions and regulation in PD are required to improve the prospects for successful treatment of PD.

## Figures and Tables

**Table 1:** Kyoto Encyclopedia of Genes and Genomes pathways enriched for Parkinson’s disease down-regulated genes, generated using the Database for Annotation, Visualization and Integrated Discovery.

KEGG Pathway	Count	%	*p*-Value	Genes
ECM-receptor interaction	10	0.120	0.007	*CD36*, *ITGA5*, *LAMA5*, *ITGA8*, *COL6A3*, *COL6A2*, *ITGA1*, *DAG1*, *RELN*, *COL5A3*
P53 signaling pathway	8	0.096	0.021	*STEAP3*, *ZMAT3*, *CD82*, *BAX*, *SHISA5*, *MDM2*, *CCNG2*, *GADD45A*
Arrhythmogenic right ventricular cardiomyopathy	8	0.096	0.037	*ITGA5*, *ITGA8*, *ITGA1*, *DAG1*, *GJA1*, *CACNB3*, *TCF7L2*, *TCF7L1*
Focal adhesion	15	0.181	0.038	*CAV1*, *TLN1*, *ITGA1*, *COL5A3*, *CAPN2*, *MYL9*, *DOCK1, LAMA5*, *ITGA5*, *ITGA8*, *COL6A3*, *COL6A2*, *PDGFRB*, *RELN*, *ZYX*
Endocytosis	14	0.168	0.041	*GIT1*, *ARFGAP1*, *CHMP2A*, *PLD1*, *ERBB3*, *RUFY1*, *ASAP3*, *EPS15*, *FAM125A*, *AP2A1*, *VPS24*, *VPS4A*, *MDM2*, *VPS28*

## Data Availability

Data is contained within the article or Supplementary Materials. The data presented in this study are available.

## Supplementary Materials

The following are available online at https://www.mdpi.com/article/10.3390/genes12071066/s1, Figure S1: Cell viability of control and Parkinson’s disease (PD) cells at passage 8 following treatment with either DPBS or various concentrations of human recombinant Reelin (hr-Reelin) protein.
