# Supplementary material for: Reelin Alleviates Mesenchymal Stem Cell Senescence and Reduces Pathological α-Synuclein Expression in an In Vitro Model of Parkinson’s Disease
Source: Genes (Basel). 2021 Jul 13;12(7):1066. doi: 10.3390/genes12071066 (PMC8308051; doi:10.3390/genes12071066)
Supplement: Supplementary file 1 [file genes-12-01066-s001.zip › genes-1192909-supplementary.pdf]

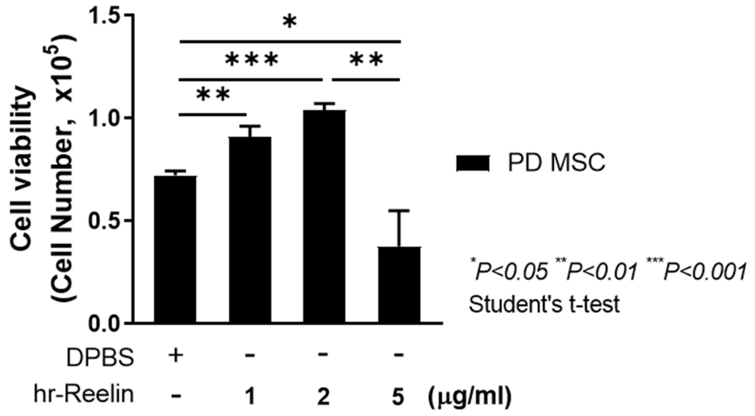

Figure S1. Cell viability of control and Parkinson's disease (PD) cells at passage 8 following treatment with either DPBS or various concentrations of human recombinant Reelin (hr-Reelin) protein. n = 12 for DPBS treated group; n = 7 for 1 μg/ml and 2 μg/ml hr-Reelin treated group; n = 8 for 5 μg/ml hr-Reelin treated group. Mean ± S.E.M. Student T-test, * $p < 0.05$; ** $p < 0.01$; ***$p < 0.001$.
